# Analysis of the transcriptome data in *Litopenaeus vannamei* reveals the immune basis and predicts the hub regulation-genes in response to high-pH stress

**DOI:** 10.1371/journal.pone.0207771

**Published:** 2018-12-05

**Authors:** Wen Huang, Hongmei Li, Chuhang Cheng, Chunhua Ren, Ting Chen, Xiao Jiang, Kaimin Cheng, Peng Luo, Chaoqun Hu

**Affiliations:** 1 CAS Key Laboratory of Tropical Marine Bio-resources and Ecology (LMB), Guangdong Provincial Key Laboratory of Applied Marine Biology (LAMB), South China Sea Institute of Oceanology, Chinese Academy of Sciences, Guangzhou, China; 2 South China Sea Bio-Resource Exploitation and Utilization Collaborative Innovation Center, Guangzhou, China; 3 University of Chinese Academy of Sciences, Beijing, China; 4 Yuehai Feed Group co., LTD, Zhanjiang, China; Chang Gung University, TAIWAN

## Abstract

Soil salinization erodes the farmlands and poses a serious threat to human life, reuse of the saline-alkali lands as cultivated resources becomes increasingly prominent. Pacific white shrimp (*Litopenaeus vannamei*) is an important farmed aquatic species for the development and utilization of the saline-alkali areas. However, little is known about the adaptation mechanism of this species in terms of high-pH stress. In the present study, a transcriptome analysis on the gill tissues of *L*. *vannamei* in response to high-pH stress (pH 9.3 ± 0.1) was conducted. After analyzing, the cyclic nucleotide gated channel-Ca^2+^ (CNGC-Ca^2+^) and patched 1 (Ptc1) were detected as the majority annotated components in the cAMP signaling pathway (KO04024), indicating that the CNGC-Ca^2+^ and Ptc1 might be the candidate components for transducing and maintaining the high-pH stress signals, respectively. The immunoglobulin superfamily (IgSF), heat shock protein (HSP), glutathione s-transferase (GST), prophenoloxidase/phenoloxidase (proPO/PO), superoxide dismutase (SOD), anti-lipopolysaccharide factor (ALF) and lipoprotein were discovered as the major transcribed immune factors in response to high-pH stress. To further detect hub regulation-genes, protein-protein interaction (PPI) networks were constructed; the genes/proteins “Polymerase (RNA) II (DNA directed) polypeptide A” (POLR2A), “Histone acetyltransferase p300” (EP300) and “Heat shock 70kDa protein 8” (HSPA8) were suggested as the top three hub regulation-genes in response to acute high-pH stress; the genes/proteins “Heat shock 70kDa protein 4” (HSPA4), “FBJ murine osteosarcoma viral oncogene homolog” (FOS) and “Nucleoporin 54kDa” (NUP54) were proposed as the top three hub regulation-genes involved in adapting endurance high-pH stress; the protein-interactions of “EP300-HSPA8” and “HSPA4-NUP54” were detected as the most important biological interactions in response to the high-pH stress; and the HSP70 family genes might play essential roles in the adaptation of the high-pH stress environment in *L*. *vannamei*. These findings provide the first insight into the molecular and immune basis of *L*. *vannamei* in terms of high-pH environments, and the construction of a PPI network might improve our understanding in revealing the hub regulation-genes in response to abiotic stress in shrimp species and might be beneficial for further studies.

## Introduction

Soil salinization is a global problem which significantly impacts crop yields and poses a serious threat to regional human life [1–4]. High-pH of alkaline soil can inhibit plant growth by decreasing nutrient solubility and lead to death of the plant [5]. It is reported that about 8.31 × 10^8^ hectares (ha) of soil resources and 20 percent of irrigated lands are threatened by the soil salinization worldwide [5–7], and more than 1.5 × 10^6^ ha of irrigated land are continuously affected by soil salinization and taken out of production each year [8–9]. With the rapid growth of human population and food security crisis in the world, reuse of the saline-alkali lands as cultivated resources becomes more and more prominent and [6], appropriate strategies for development and utilization of the saline-alkali territorial resources are needed to be exploited.

China is one of the countries whose lands are severely affected by soil salinization (the area is about 3.6 × 10^7^ ha) [6–7, 10]. The aquaculture industries, introduced to these areas in recent years in the country, are now improving the productivity and ecological environment in the salinization regions [11–12]. Liu et al. (2016) estimated that the output value of aquaculture industries in saline-alkali waters reached $1.14 million US in Cangzhou City of Hebei Province of China [12]. By introducing the aquaculture industries to Dali County (Weinan City, Shaanxi Province, China), the top pH value of saline-alkali lands in the areas drops from 9.01 to 8.56 [13]. Pacific white shrimp (*Litopenaeus vannamei*, *L*. *vannamei*), naturally inhabiting in the tropical environments of the Western Pacific coast, is a dominant crustacean farmed worldwide whose production is up to 2,721,929 tons (more than $11 billion US) in 2010 [14–18]. With a strong tolerance to salinity (0.5 to 45 ppt) [19–20], good resistance to stress [21–22] and high economic value of this species [23], *L*. *vannamei* has become one of the most important aquatic species for culturing in saline-alkali water areas [24–26].

RNA-Seq technology is a useful method for revealing the regulation profiles of non-model species [17, 27–28]. After proper transcriptome assembly, large-scale genes of non-model species can be defined by comparing with other model species, and that would be of value to make extensive functional annotation publicly available to the scientific community [18]. The expression profiles of *L*. *vannamei* in various stress conditions, such as high salinity [29], low salinity [30–33], nitrite [34] and ammonia stress [15], have already been revealed through RNA-Seq technology. The transcriptome analysis of long-term adaptation under chronic low-salinity stress is conducted in *L*. *vannamei*, and the study improves our understanding of the mechanisms underlying osmoregulation in euryhaline crustaceans [31]. Transcriptome of nitrite-exposed *L*. *vannamei* identified numerous candidate genes associated with immune response, detoxification, apoptosis pathways that might be involved in response to nitrite stress [34]. The transcriptome analysis in the hepatopancreas of *L*. *vannamei* under acute ammonia stress supplies the molecular mechanism of the immunosuppression from ammonia stress [15]. In spite of the important utilization of farming industry in the saline-alkali waters, transcriptome studies involving *L*. *vannamei* in high-pH environments are still lacking.

Generally, hundreds of candidate differentially expressed genes (DEGs) are unavoidably obtained after filtering and analyzing the RNA-Seq data in non-model species or in organisms that lack whole-genome sequencing [15, 17, 35]. The protein–protein interactions (PPIs), which are the central relationship in most biological activities [36], are available for revealing the associations among genes [37]. Important genes and related pathways can be identified by constructing PPI networks, as can be observed in the report of Zang et al. (2018), four predominant genes in uterine leiomyosarcoma (uLMS) are identified after analyzing the PPI networks of 95 differentially expressed genes [38]. The “Betweenness” of an actor *i* is defined as the total number of shortest paths between pairs of actors that pass through *i* and is an indicator of the most influential factors in the network [39–40]. The algorithm of “Betweenness” is an effective method for analyzing the candidate hub genes in PPI networks. Karbalaei et al. (2018) analyzed Alzheimeir’s disease (AD) and non-alcoholic fatty liver disease (NAFLD) using PPI networks, and, based on the Betweenness values, seven hub-bottleneck node genes were discovered [41].

The balance of acid-alkali ions in water is of essential importance for aquatic crustaceans [42–46]. The decreasing pH value in seawaters caused by ocean acidification (OA) [47] negatively affects the mortality, growth, and reproduction of marine crustaceans [48–50]. Kurihara et al. (2008) evaluated that the survival rates of *Palaemon pacificus* were largely reduced (55%) after long-term (30 weeks) exposing to low-pH environment (pH 7.89 ± 0.05) [51]. Gao et al. (2018) reported that composition of the fatty acids (monounsaturated fatty acids, polyunsaturated fatty acids, eicosapentaenoic acid and docosahexaenoic acid), which were well known to be involved in immune modulatory effects, were significantly affected by low-pH environment (pH 7.6–7.8) in brine shrimp *Artemia sinica* [52]. The immunity of *L*. *vannamei* has been demonstrated to be influenced after being exposed to high-pH stress [53–54]. The report of Li and Chen (2008) showed that several immune parameters including phenoloxidase (PO) activity, superoxide dismutase (SOD) activity and total haemocyte count (THC) were significantly decreased after transferring the shrimp to high-pH stress environments, which led to decrease the resistance of *L*. *vannamei* against *Vibrio alginolyticus* [53]. When *L*. *vannamei* were exposed to acute stress, with pH 5.6 and pH 9.3, oxidative damage occurred by increasing the reactive oxygen species (ROS) production and the DNA comet assay value [54]. While Sookruksawong et al. (2013) proved that the PO pathway genes were also associated with resistance to Taura syndrome virus (TSV) in *L*. *vannamei* [55], Zhang et al. (2007) elucidated that the expression of SOD gene changed rapidly and dynamically in response to white spot syndrome virus (WSSV) in shrimp *Fenneropenaeus chinensis* [56]. These results indicated that the farming of *L*. *vannamei* in high-pH environments might weaken the immunity of shrimp to pathogens, and probably affect the final production.

Molecular signaling pathways can help to elucidate the activity and coordination of the cell-expression network in response to extracellular stresses [17], which is concerned to play key roles in revealing the regulation pattern of innate immune system in shrimp [57–58]. The important types of signaling genes, such as calcium/calmodulin-dependent protein kinases [59], mitogen-activated protein kinases (MAPKs), transcription factor (TF) and serine/threonine-protein kinases [60–61], are recognized as the key components for converting extracellular stimuli into a wide range of cellular responses in organisms, from yeast to humans [4, 27, 59–61]. The understanding of the regulatory features of the immune factors and signaling genes involved in high-pH environmental adaptation in *L*. *vannamei* will be beneficial and necessary in further studies.

In the present study, high-pH RNA-Seq was carried out in the gill tissues of *L*. *vannamei* and the major types of signaling genes, regulation pathways and immune factors were investigated. The PPI networks of the major differentially expressed genes were constructed, and the candidate hub regulation-genes were predicted. Our results will be helpful for revealing a deeper insight into the molecular basis for the adaptation of *L*. *vannamei* to a high-pH environment.

## Materials and methods

### Treatments of the shrimp samples

**Fig 1:**
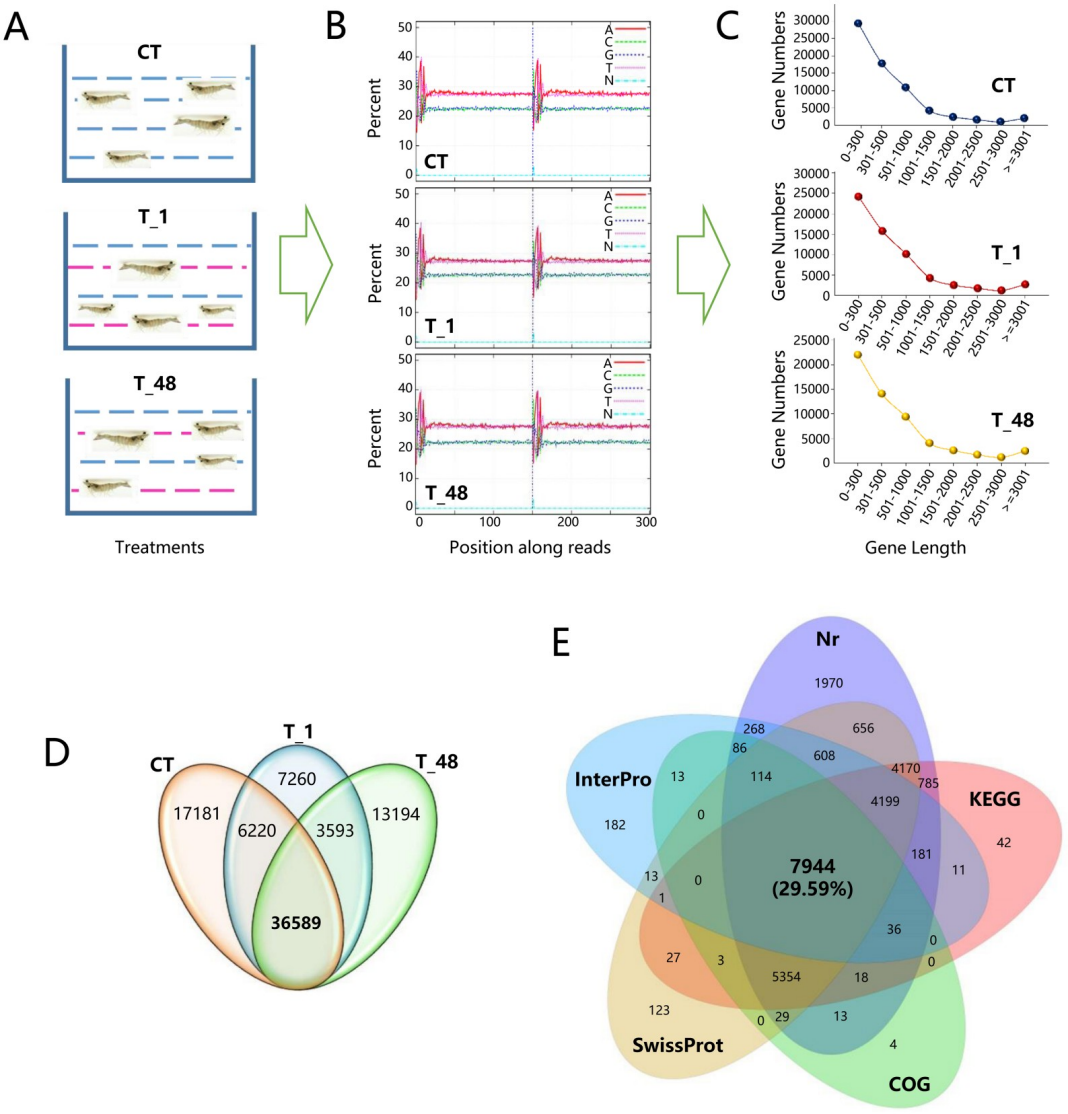
Overview of the basic sequencing data. (**A**) Treatments of the three groups of shrimp materials. “CT” represented the control group (pH 8.0 ± 0.1). Red dotted lines indicated the stress environments of pH 9.3 ± 0.1; the shrimps were treated either for 1 hour (T_1) or 48 hours (T_48). (**B**) Quality control of the three libraries. The distributions of the base compositions on the clean reads were indicated by different lines next to the upper right corner. X axes represented the base position along the reads. Y axes represented base content percentages. The conspicuous fluctuations in the first six base positions of each contig (150 bp length) were the normal situation and were caused by using the random hexamer-primer to synthesize the cDNA. (**C**) Unigene length distribution. X axes represented the length of the Unigenes and Y axes represented the number of Unigenes. (**D**) The Venn diagram of gene numbers distributed among the three libraries. (**E**) Venn diagram of the annotated gene numbers in the Nr, KEGG, COG, SwissProt and Interpro databases.

According to Wang et al. (2009), oxidative damage (ROS production and DNA damage increased in the haemocytes and the hepatopancreas cells) occurred when the shrimps were exposed in pH 9.3 stress environment [54]. In this study, healthy reared shrimps (supplied by Donghai Island Department, Yuehai Feed Group co., LTD, Zhanjiang, China) with an average weight of 11.09 ± 2.37 g were selected, three groups were placed in control seawater (CT group, pH 8.0 ± 0.1, 3.0% salinity, 28 °C temperature), in pH 9.3 ± 0.1 seawater for 1 hour (T_1 group, 3.0% salinity, 28 °C temperature) and in pH 9.3 ± 0.1 seawater for 48 hours (T_48 group, 3.0% salinity, 28 °C temperature) (Fig 1A), each group contained 20 shrimps and cultured in the same separate tank (50 L of seawater). The pH environments in T_1 and T_48 group were raised and maintained to 9.3 ± 0.1 by the gradual addition of 1 mol/L each of Na_2_CO_3_ and NaHCO_3_ in the original tank, pH was monitored over the subsequent 1 hour and 48 hours using a 228573-A01 pH electrode (Orion Research Inc., Beverly, MA, USA) attached to an Orion320 pH meter [54].

The gill tissues were separated and were immediately stored in liquid nitrogen. Six biological replicates were obtained from each group, 50–100 mg of gill tissues were extracted by 1 mL TRIzol Reagent (Invitrogen, USA) according to the manufacturer’s instructions for each individual, 1 μg RNA of each individual were gained, 6 μg RNA from the six biological replicates were pooled in each group and were used for the subsequent experiments [17]. All the experiments were conducted in South China Sea Institute of Oceanology, Chinese Academy of Sciences, Guangzhou, China.

### RNA isolation and cDNA library construction

Three separate libraries (CT, T_1 and T_48) were sampled. The following steps were described by Huang et al. (2017) [17]. First, the poly-A tailed mRNA was concentrated by oligo (dT)-coated magnetic beads; fragmentation was performed by incubation in an NEB Next First Strand Synthesis Reaction Buffer (NEB, USA); the second strand was generated with the buffer, dNTPs, RNase H and DNA polymerase-I; and the adapters were ligated to the synthesized cDNA fragments after an end repair step. The Illumina HiSeq 4000 sequencing platform (paired-end 150 bp reads were generated) (BGI Company, Shenzhen, China) was employed to construct the libraries (CT, T_1 and T_48).

The detailed assembly methods of the transcripts were described in the reports of Li et al. (2017) [62] and Zhang et al. (2018) [63], briefly: the raw sequence data were filtered with SOAPdenovo software [64–65], adaptor-polluted, low-quality (A Phred quality score is used to identify the quality of the reads) [66–67] and high content of unknown base (N) reads were removed; after filtering, Trinity software (parameters:—min_contig_length150—CPU 8—min_kmer_cov 3—min_glue3—bfly_opts ‘-V 5—edge-thr = 0.1—stderr’) [68] was employed to perform de novo assembly with the clean reads, and these were first joined to Contigs and then connected to sequences. The sequences that could not be extended were defined as Unigenes. TIGR Gene Indices clustering tool (parameters: -l40-c 10-v 25-O ‘-repeat_stringency 0.95-minmatch35-minscore35’) [69] was used to obtain the final Unigenes for further analysis [62].

### Quantification of the differentially expressed genes (DEGs)

Two comparisons of T_1/CT and T_48/T_1 were applied among the three libraries (CT, T_1 and T_48). The DEGs in comparison between T_1/CT represented the transcripts in response to an acute stress pH environment. The DEGs in the comparison between T_48/T_1 represented the transcripts in response to the endurance pH environment (endured high-pH for 48 hours). The metric fragments per kilobase of transcript per million mapped reads (FPKM) value was used to estimate transcript abundance on the basis of eliminating the influence of different gene lengths and sequencing discrepancies [70]. Therefore, the FPKM values were directly used to compare the differences in gene expressions among the samples. The algorithm developed by Audic and Claverie (1997) was used to compare the differences in various gene expression levels [71]. A value of the FDR (false discovery rate) ≤ 0.001 and an absolute value of the Log2 ratio ≥ 1.0 were set as the criteria values. The coexisted DEGs in the two comparisons were filtered, the Log_10_^FPKM^ values were used to represent the expression values of transcriptional libraries (CT, T_1 and T_48), and the software Multiple Experiment Viewer (MeV 4.9.0) [72] was employed to analyze the expression profiles of the large numbers of coexisted DEGs.

### Identification of the major pathways

After assembly, the transcript-genes were annotated by gene ontology terms (GO) [73], Kyoto Encyclopedia of Genes and Genomes Pathway (KEGG) [74], Non-redundant NCBI collection of nucleotide (NT) and protein (NR) sequence database (ftp://ftp.ncbi.nih.gov/blast/db/FASTA/), the Swiss-Prot database (SwissProt) [75] and the Cluster of Orthologous Groups (COG, http://www.ncbi.nlm.nih.gov/COG/) protein database [76–77].

### Identification of the major types of immune factors and signaling genes

According to previous studies, 17 types of immune factors including immunoglobulin superfamily (IgSF) [78–79], prophenoloxidase/phenoloxidase (proPO/PO) [53, 80], thioredoxin (TRx) [54], heat shock protein (HSP) [78, 81], superoxide dismutase (SOD) [53–54, 82], glutathione peroxidase (GPx) [54, 82–83], anti-lipopolysaccharide factor (ALF) [84], chitin-binding protein (CBP) [85], down syndrome cell adhesion molecule (Dscam) [86], antimicrobial peptides (AMPs) [85–86], toll-like (TL) [78], peroxinectin (PX) [80], glutathione s-transferase (GST) [87–88], lipoprotein [89], lysozyme [82], catalase [54, 82–83] and penaeidins [85] were selected from the total annotated genes. The calcium and MAPK signaling genes, transcription factor and serine/threonine-protein signaling genes were searched among the total annotated genes [17, 27].

The major types of immune factors and signaling genes from the two comparisons were filtered by a P-value < 0.05, a FDR ≤ 0.001 and an absolute value of Log_2_^FoldChange^ ≥ 3.0. The Log_10_^FPKM^ values were used to represent the expression values of transcriptional libraries (CT, T_1 and T_48) and to create heat maps with the software Heatmap Illustrator (Heml 1.0.3.3) [90].

### Analysis of the PPI network for the major DEGs

Major DEGs from the two comparisons were obtained by a P-value < 0.05, -Log_10_^FDR^ ≥ 10.0 and an absolute value of Log_2_^FoldChange^ ≥ 3.0. The PPI networks of the major DEGs were constructed as Szklarczyk et al. (2011) [91] and Yang (2018) [92] described previously. Simply, the major DEGs were annotated by the SwissProt database, the annotated proteins were submitted to the STRING database (http://string-db.org/) for searching the experimental and interaction information, the detected proteins and interactions were exported and conserved as a file of simple tabular text output (tab separated values, TSV), and the criteria of the combined score was set to no less than 0.6 to keep the network analysis of PPI manageable [93]. The PPI network was visualized with Cytoscape version 3.4.0 software (http://cytoscape.org/) [94], the protein nodes were attributed to forming the circle layout, and the detailed user manual of the software was listed in the website http://www.gnu.org/licenses/lgpl-2.1.html. To analyze the candidate hub genes and interactions, the algorithm of “EdgeBetweenness” was employed to calculate the PPI networks [39–40], and the EdgeBetweenness values were obtained with Cytoscape version 3.4.0 software [94].

### Real-time PCR validation

To verify the expression profiles of genes in our RNA-Seq results, the represented 10 DEGs in the two comparisons were selected for validation of the Illumina sequences by real-time PCR analysis. All the primers used were listed in S1 Table. The treatments that were performed were described above, and the relative transcript abundance was obtained by normalizing with the expression of the *L*. *vannamei* β-actin gene based on the 2^-ΔΔCT^ method [17, 95].

## Results

### Overview of the RNA-Seq data

After filtering, the distribution of the base composition on clean reads was monitored, all the adenine bases (A) were detected to overlap with the thymine bases (T), and all the guanine bases (G) were observed to overlap with the cytosine bases (C) (Fig 1B). The total clean reads per library ranged from 44.04 Mb to 44.58 Mb, and the clean reads ratio ranged from 82.08% to 83.98% (Table 1). The clean reads of the three libraries (CT, T_1 and T_48) were deposited at the US National Center for Biotechnology Information (NCBI) Sequence Read Archive (SRA, http://www.ncbi.nlm.nih.gov/Traces/sra) with the GenBank accession No. SRP162932. The clean reads were assembled and clustered into Unigenes, the total number of Unigenes was 78,080 (Table 2), the most abundant Unigene length were clustered in the group of 0–300 bp (Fig 1C), and the coexisted Unigene number for the three libraries was 36,589 (Fig 1D). Overall, the Unigenes returning hits in Nr, KEGG, COG, Swiss-Prot and InterPro databases were 26,850, and the compositions for each database were 26,431 (98.44%), 22,771 (84.81%), 13,614 (50.70%), 23,241 (86.56%), and 13,656 (51.38%), respectively (Fig 1E). The coexisted gene number of the five databases was 7,944 (which occupied 29.59% of all annotated genes) (Fig 1E). The complete nucleotide sequences of each Unigene were available in S1 File.

**Table 1:** Summary of quality metric reads after filtering.

Sample	Total Raw Reads (Mb)	Total Clean Reads (Mb)	Total Clean Bases (Gb)	Clean Reads Q20^a^ (%)	Clean Reads Q30^b^ (%)	Clean Reads Ratio (%)
CT	53.66	44.04	6.61	98.43	95.55	82.08
T_1	63.62	44.58	6.69	98.39	95.43	83.15
T_48	53.70	45.09	6.76	98.56	95.79	83.98

^a^ Q20: the rate of bases which Phred base-calling quality by illumine is greater than 20; and

^b^ Q30: The rate of bases which Phred base-calling quality by illumine is greater than 30.

**Table 2:** Quality metrics of Unigenes.

Sample	Total Number	Total Length	Mean Length	N50^a^	N70^b^	N90^c^	GC(%)^d^
CT	69,643	45,596,560	654	1,130	493	257	42.73
T_1	62,677	46,992,121	749	1,431	624	275	42.71
T_48	57,534	43,546,433	756	1,451	647	276	42.68
All-Unigene	78,080	54,187,520	694	1,349	529	256	42.46

^a^ N50: a weighted median statistic that 50% of the Total Length is contained in Unigenes great than or equal to this value;

^b^ N70: a weighted median statistic that 70% of the Total Length is contained in Unigenes great than or equal to this value;

^c^ N90: a weighted median statistic that 90% of the Total Length is contained in Unigenes great than or equal to this value.

^d^ GC (%): the percentage of G and C bases in all Unigenes.

### Quantification review of the DEGs

**Fig 2:**
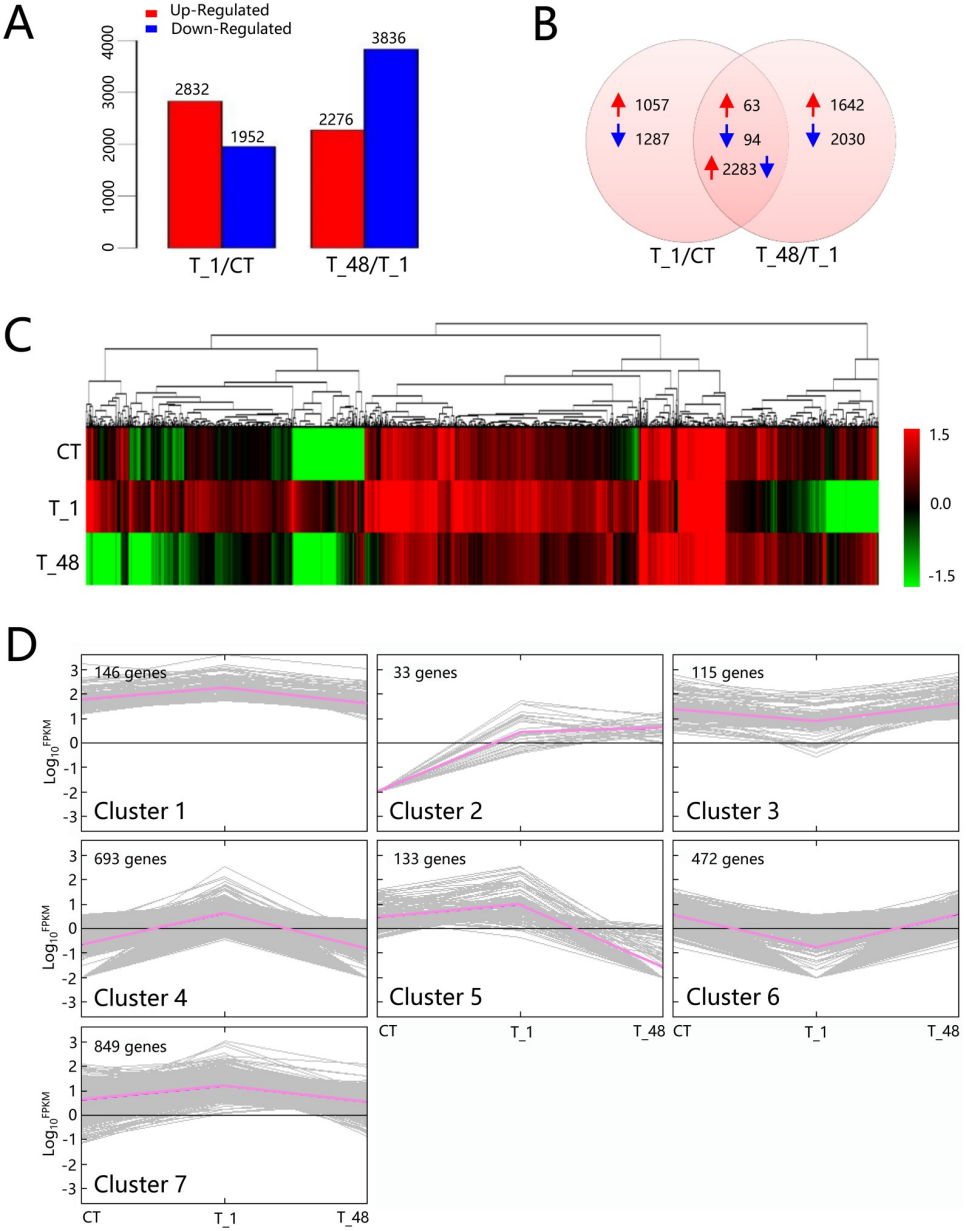
Quantification and expression profiles of the DEGs. (**A**) Column statistics of DEG numbers in the two comparisons. (**B**) Venn diagram of the up- and down-regulated DEGs. Red arrows represented the up-regulated genes. Blue arrows represented the down-regulated genes. (**C**) Profiling heatmap of the coexisted DEGs. The Log_10_^FPKM^ values of the coexisted DEGs from the expression libraries of CT, T_1 and T_48 were used to construct the heatmap. (**D**) Classification of the 2,440 coexisted DEGs from the expression libraries of CT, T_1 and T_48. Gene numbers were noted in the upper left corners. The red bold lines represented the average profile in each cluster. Gray lines indicated the expression profiles in the CT, T_1 and T_48 libraries.

A total of 2,832 up- and 1,952 down-regulated genes were detected in the comparison of T_1/CT (Fig 2A), and 2,276 up- and 3,836 down-regulated genes were discovered in the comparison of T_48/T_1 (Fig 2A). A total of 2,440 (63 + 94 +2283) coexisted DEGs (occupied 28.86% of all DEGs) were detected, 63 and 94 genes were observed to be up-regulated or down-regulated, respectively, in both T_1/CT and T_48/T_1 comparisons (Fig 2B). To investigate the expression profiles, the 2,440 coexisted DEGs were clustered and classified into 7 gene clusters (Hierachical clustering was analyzed by Euclidean distance, the gene tree was classified by the distance threshold ranged from 2.30–2.50) (Fig 2C and 2D), of which, the top three clusters were detected (Cluster 7, Cluster 4 and Cluster 6), and the gene numbers of the top three clusters contained 82.54% of the total coexisted DEGs (Fig 2). The expression profiles of the top three cluster genes were profoundly influenced in the T_1 group and recovered to nearly the control level thereafter in the T_48 group (Fig 2D), which indicated that the expression level of the majority of the regulated genes in shrimp returned to normal conditions after long-term exposure to the pH 9.3 environment.

### GO and COG analysis of the DEGs

**Fig 3:**
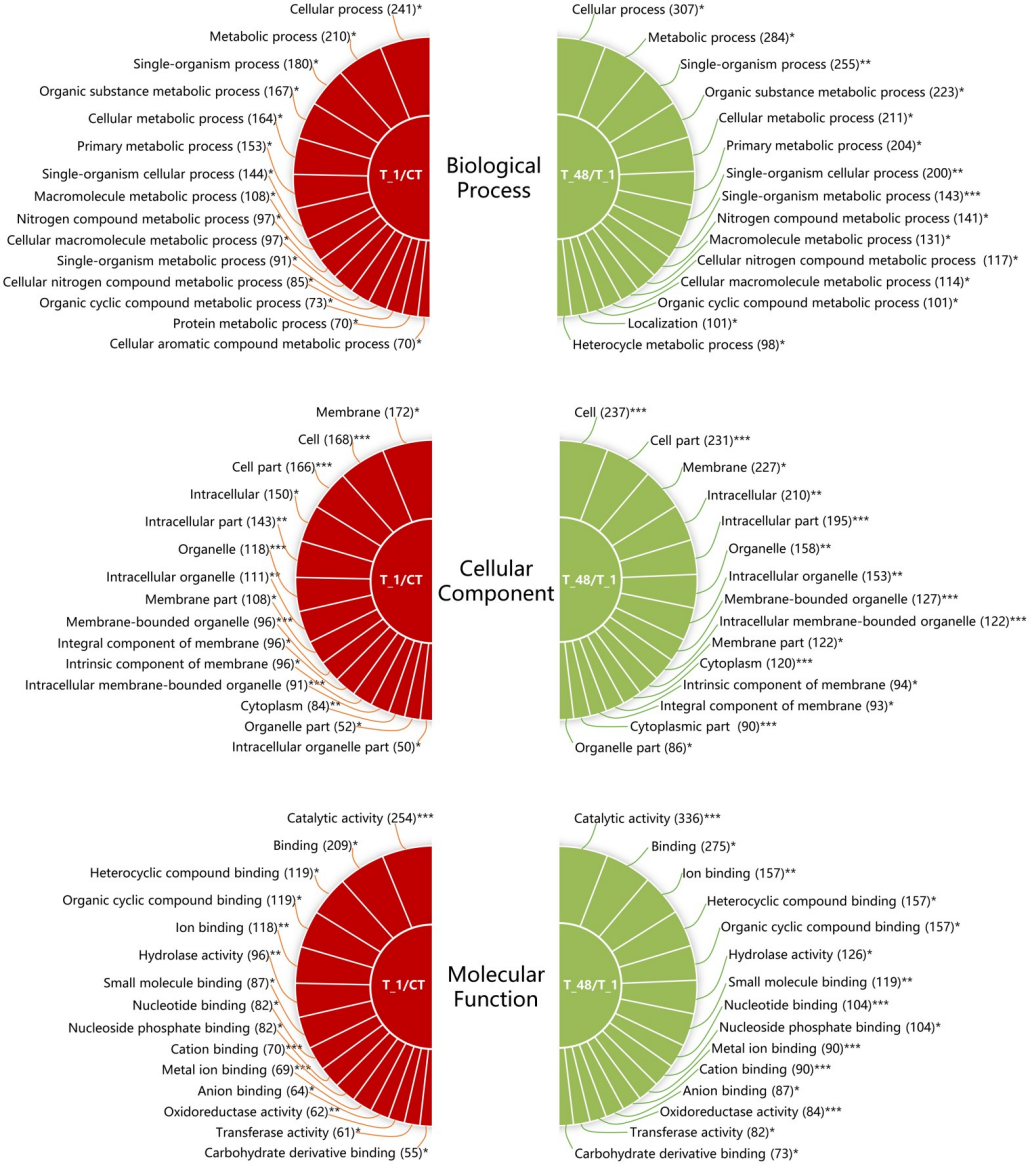
Gene ontology results of the biological process, cellular component and molecular function in the two comparisons of T_1/CT and T_48/T_1. GO terms and the associated gene numbers in the comparison groups of T_1/CT and T_48/T_1 were indicated. “*” indicated that the corrected P-value of the GO terms ranged from 0.5–1.0. “**” indicated that the corrected P-value of the GO terms ranged from 0.1–0.5. “***” indicated that the corrected P-value of the GO terms was lower than 0.1. The lower the value of the corrected P-value, the more credible the term.

After analyzing, the biological process was detected as the most annotated GO category in both comparisons of T_1/CT (329 DEGs) and T_48/T_1 (448 DEGs) (Table 3). The top 15 clustered GO terms in categories of biological process, cellular component and molecular function were summarized in both the two comparison groups, respectively (Fig 3). Take the category of biological process for example, cellular process (GO:0009987), metabolic process (GO:0008152), single-organism process (GO:0008150), organic substance metabolic process (GO:0071704) and cellular metabolic process (GO:0044237) were discovered as the top 5 annotated GO terms for both libraries comparisons (Fig 3). Specifically, 241 DEGs were annotated to GO term of cellular process in the comparison group of T_1/CT and 307 genes were annotated to the same GO term in the comparison group of T_48/T_1 (Fig 3). The membrane (GO:0016020) was the most annotated GO term of the cellular component category in the T_1/CT comparison group, and the cell (GO:0005623) was the most annotated GO term in the T_48/T_1 comparison group (Fig 3). The catalytic activity (GO:0003824) was the most annotated GO term of the molecular function category in both the T_1/CT and T_48/T_1 comparison groups (Fig 3).

**Table 3:** Summary of gene ontology analysis.

Comparison	Category	Number of annotated gene	Number of annotated GO term
T_1/CT	Biological process	329	697
	Cellular component	291	122
	Molecular function	370	222
T_48/T_1	Biological process	448	871
	Cellular component	403	201
	Molecular function	506	308

The COG data of the DEGs were summarized, the top three functional categories of “General function prediction only” (657 DEGs were clustered), “Translation, ribosomal structure and biogenesis” (588 DEGs were clustered) and “Carbohydrate transport and metabolism” (337 DEGs were clustered) were detected (Table 4), and the detailed DEGs information for COG annotation was available in S2 Table.

**Table 4:** Cluster of orthologous groups of the DEGs.

Functional categories	Represented code	Number of clustered DEGs
General function prediction only	R	657
Translation, ribosomal structure and biogenesis	J	588
Carbohydrate transport and metabolism	G	337
Posttranslational modification, protein turnover, chaperones	O	271
Function unknown	S	259
Inorganic ion transport and metabolism	P	233
Replication, recombination and repair	L	217
Amino acid transport and metabolism	E	210
Cell cycle control, cell division, chromosome partitioning	D	208
Transcription	K	204
Signal transduction mechanisms	T	141
Cell wall/membrane/envelope biogenesis	M	126
Energy production and conversion	C	123
Secondary metabolites biosynthesis, transport and catabolism	Q	106
Lipid transport and metabolism	I	96
Coenzyme transport and metabolism	H	88
Intracellular trafficking, secretion, and vesicular transport	U	67
Cytoskeleton	Z	60
Nucleotide transport and metabolism	F	54
Chromatin structure and dynamics	B	30
Cell motility	N	14
Defense mechanisms	V	10
RNA processing and modification	A	8
Extracellular structures	W	4
Nuclear structure	Y	1

### Identification of the major KEGG pathways

**Fig 4:**
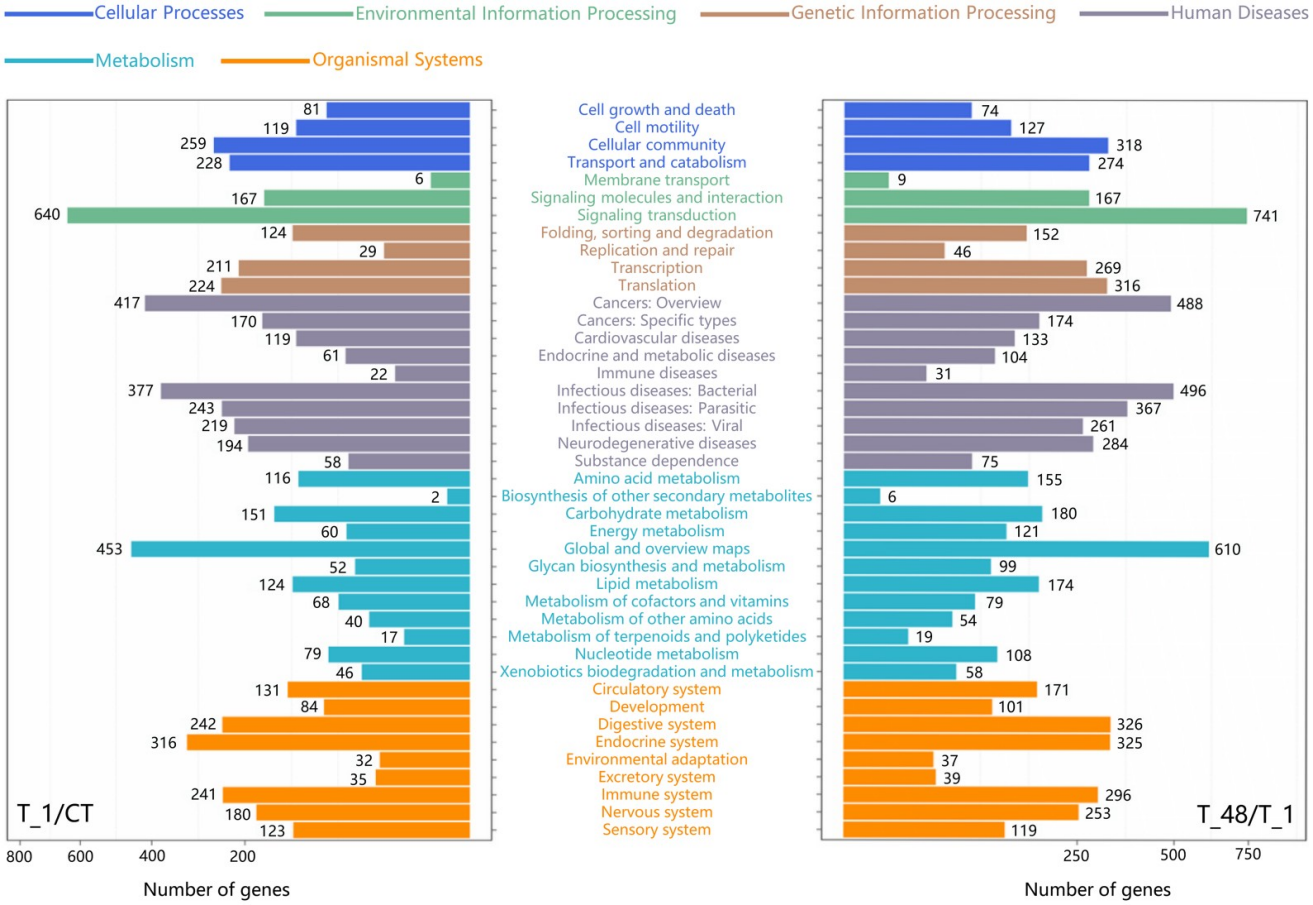
Classification and summary of the KEGG pathways in the two comparisons. Various colors indicated the KEGG categories; blue color: Cellular processes; forestgreen color: Environmental information processing; sienna color: Genetic information processing; slategray color: Human diseases; deepskyblue color: Metabolism; orange color: Organismal systems. X axes represented the annotated gene numbers.

**Fig 5:**
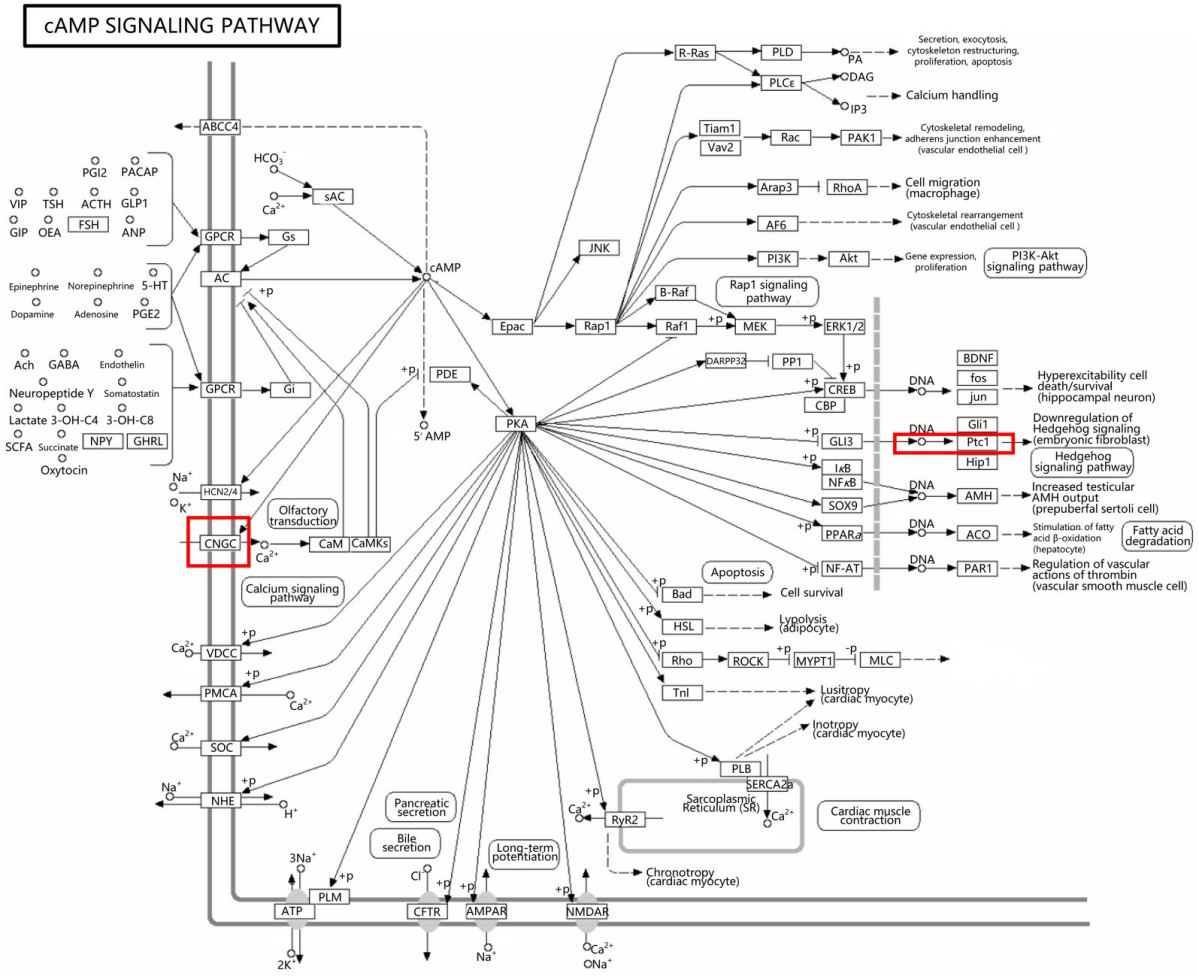
KEGG map of the cAMP signaling pathway. The red boxes framed the most annotated KOs components in the pathway.

The signal transduction pathway was the most annotated Level-2 KEGG term in both the two comparisons (T_1/CT and T_48/T_1), 640 DEGs were annotated to signal transduction in the comparison group of T_1/CT, and 741 were annotated in comparison of T_48/T_1 (Fig 4). The cAMP signaling (KO04024), cGMP-PKG signaling (KO04022) and PI3K-Akt signaling pathways (KO04151) were the top three pathways with more KOs identified in their composition regarding the two comparison groups (S3 Table). The KEGG map and DEGs of the cAMP signaling pathway were traced. The majority DEGs in the cAMP signaling pathway were annotated to the components of cyclic nucleotide gated channel beta 1 (CNGCB1) (K04952) and patched 1 (Ptc1) (K06225) in both the T_1/CT and T_48/T_1 comparisons (S4 Table), indicated that the components of CNGCB1 and Ptc1 might be the major two KOs of the annotated components in cAMP signaling pathway (Fig 5; S4 Table).

### Transcriptional profiles of the major types of immune factors and signaling genes

**Fig 6:**
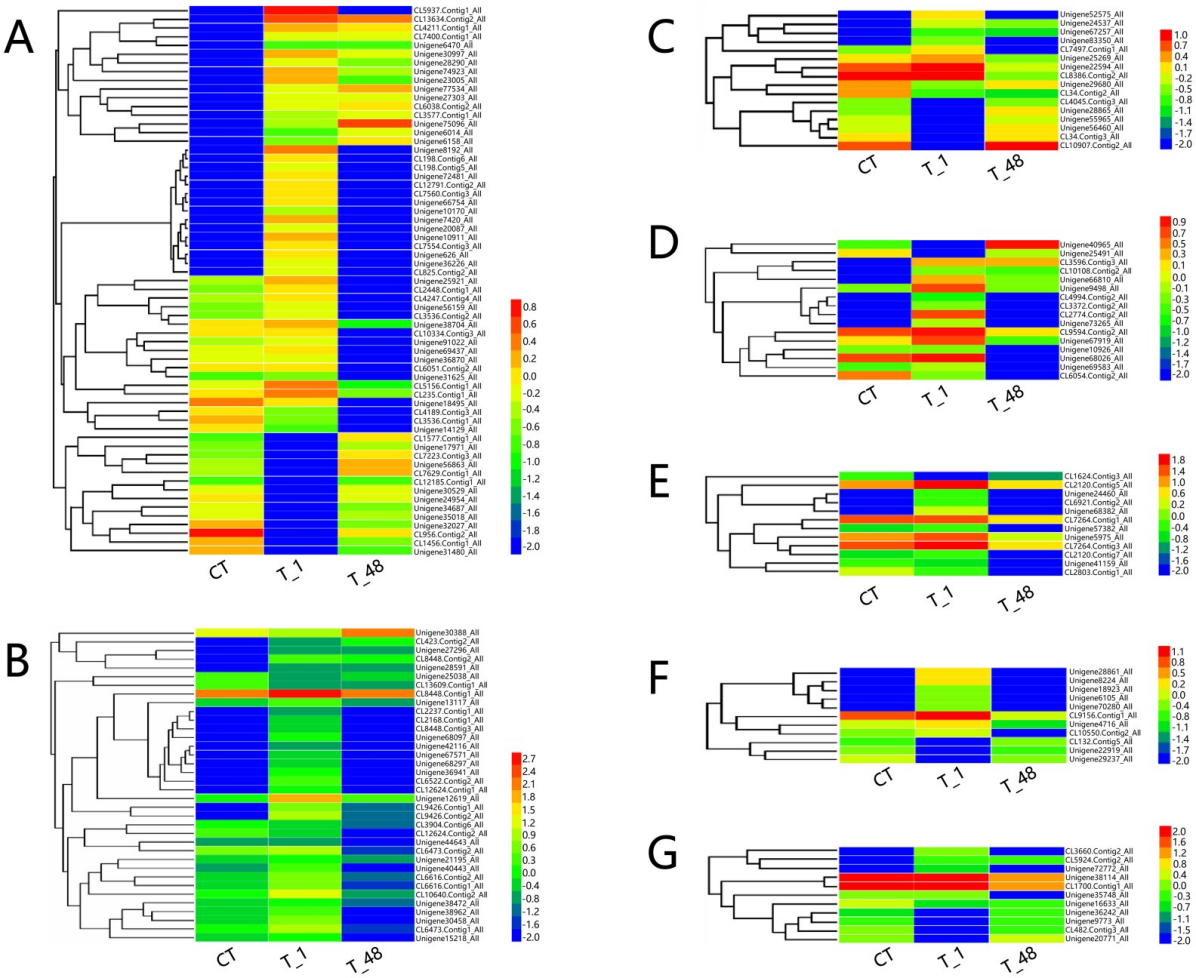
Heat maps of the major types of immune factors in *L*. *vannamei* in response to high-pH stress. (**A**): Hierarchical clustering analysis of the major annotated DEGs of “immunoglobulin superfamily”. (**B**): Hierarchical clustering analysis of the major annotated DEGs of “heat shock protein”. (**C**): Hierarchical clustering analysis of the major annotated DEGs of “glutathione s-transferase”. (**D**): Hierarchical clustering analysis of the major annotated DEGs of “prophenoloxidase/phenoloxidase”. (**E**): Hierarchical clustering analysis of the major annotated DEGs of “superoxide dismutase”. (**F**): Hierarchical clustering analysis of the major annotated DEGs of “anti-lipopolysaccharide factor”. (**G**): Hierarchical clustering analysis of the major annotated DEGs of “lipoprotein”.

After analyzing, IgSF (annotated gene number: 63), HSP (annotated gene number: 36), GST (annotated gene number: 16), proPO/PO (annotated gene number: 16), SOD (annotated gene number: 13), ALF (annotated gene number: 11) and lipoprotein (annotated gene number: 11) were detected as the top 7 types of major transcribed immune factors involved in the high-pH environment of *L*. *vannamei* (Fig 6; S5 Table). Taking the Unigene of Unigene75096_All (Gene description: Immunoglobulin G-binding protein A) as an example, the expression values were up-regulated gradually in the groups of CT (FPKM value = 0.01), T_1 (FPKM value = 0.58) and T_48 (FPKM value = 4.21) (Fig 6A; S5 Table). Among the heat shock proteins, the most up-regulated Unigene in the comparison group of T_1/CT was CL9426.Contig2_All (gene description: heat shock protein 70), the Log_2_^FoldChange^ value = 10.08. Among all the major immune factors, the CL8448.Contig1_All (gene description: heat shock protein 90) contained the highest expression value (FPKM value = 1272.53) in the T_1 group, and the Unigene30388_All (Gene description: Heat shock protein 67B2) contained the highest expression value (FPKM value = 203.54) in the T_48 group (Fig 6B; S5 Table).

**Fig 7:**
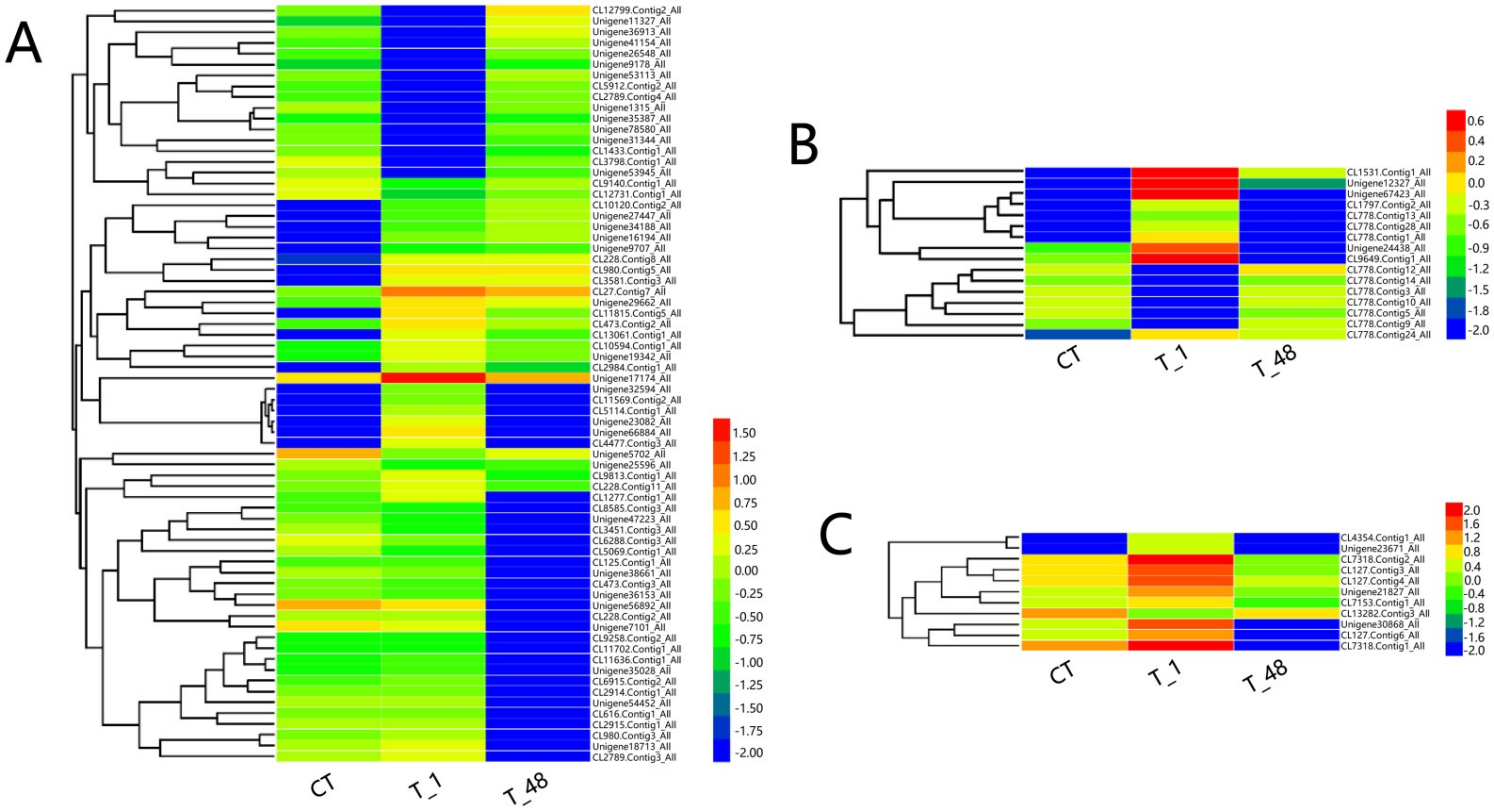
Heat maps of the major types of signaling genes in *L*. *vannamei* in response to high-pH stress. (**A**): Hierarchical clustering analysis of the major annotated DEGs of the “transcription factor”. (**B**): Hierarchical clustering analysis of the major annotated DEGs of “serine/threonine-protein kinase signaling genes”. (**C**): Hierarchical clustering analysis of the major annotated DEGs of “calcium signaling genes”.

The transcription factors (annotated gene number: 70), serine/threonine-protein kinase signaling genes (annotated gene number: 16) and calcium signaling genes (annotated gene number: 11) were selected from the total annotated genes (S6 Table), while the MAPK genes were not detected in the results, indicating that the transcription of the MAPK signaling genes would not be widely induced by the high-pH stress environment in *L*. *vannamei*. Among the transcription factors, the Unigene17174_All (gene description: Fli-1 proto-oncogene, ETS transcription factor) had the highest expression value (FPKM value = 54.09) in T_1 group, the Log_2_^FoldChange^ value of T_1/CT = 3.42 (Fig 7A; S6 Table). The calcium signaling gene of CL7318.Contig1_All (gene description: sarcoplasmic calcium-binding protein) occupied the highest expression value (FPKM value = 343.68) in the T_1 group compared with the other two types of signaling genes, the Log_2_^FoldChange^ value of T_1/CT = 3.17 (Fig 7C; S6 Table).

### PPI network of the major DEGs

**Fig 8:**
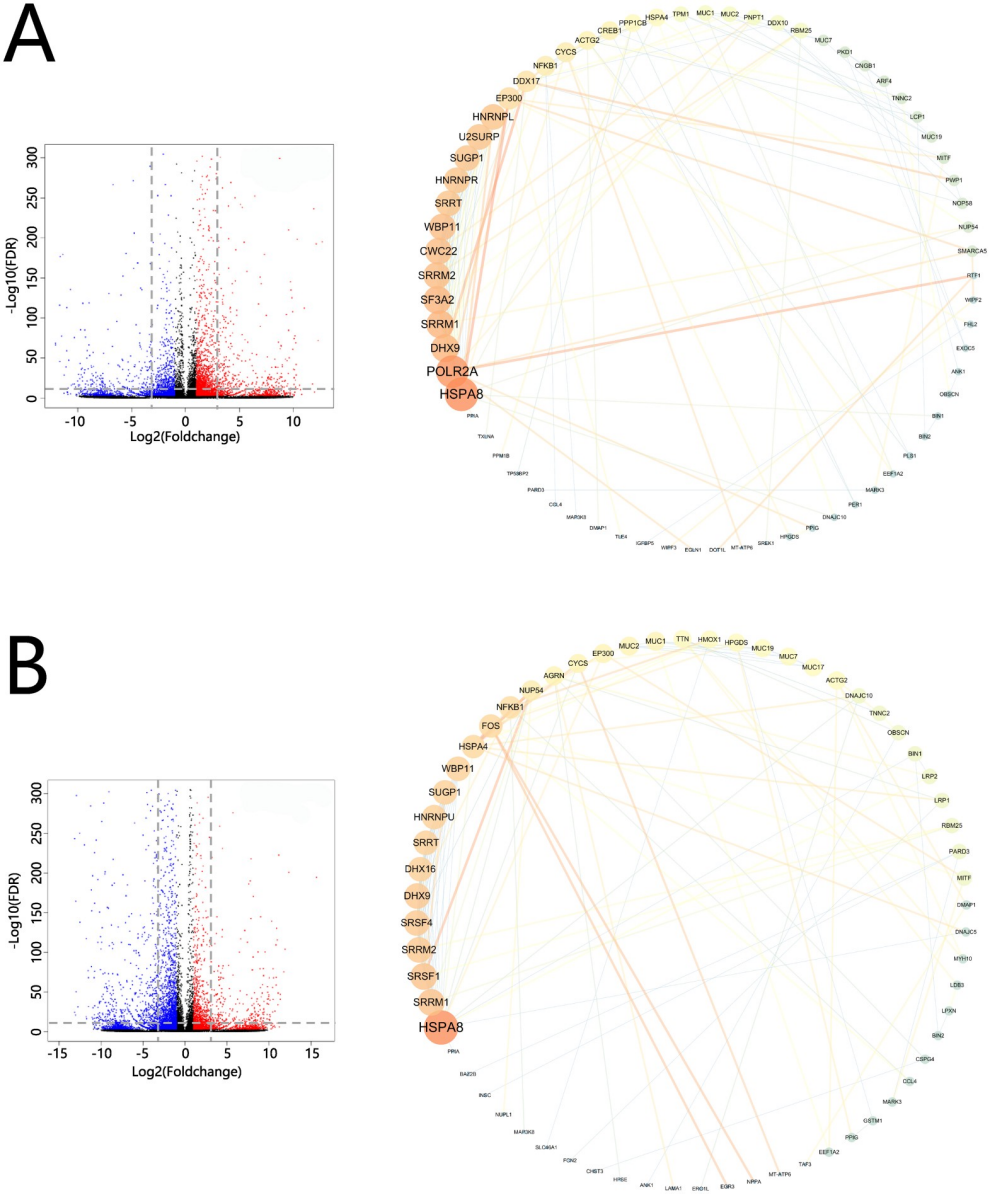
Protein-protein interaction networks of the major DEGs. (**A**) Volcano-plot figures of the DEGs in the comparison groups of T_1/CT (the left side), PPI networks of the major DEGs of T_1/CT comparison (the right side). (**B**) Volcano-plot figures of the DEGs in the comparison groups of T_48/T_1 (the left side), PPI networks of the major DEGs of T_48/T_1 comparison (the right side). On the left side of this Figure: plots indicated the expressed genes; the red color referred to up-regulated DEGs; the blue color represented down-regulated DEGs; the black plots indicated non-DEGs (absolute value of Log_2_^FoldChange^ ≤ 1.0); X axes represented the values of Log_2_^FoldChange^; Y axes represented the values of |-Log_10_^FDR^|; the higher the value on the Y axes, the more credible the value of the DEG; and the gray dash lines indicated the filter lines for the major DEGs (P-value < 0.05, -Log_10_^FDR^ ≥ 10.0, absolute value of Log_2_^FoldChange^ ≥ 3.0). On the right side of this figure: the nodes represented the genes/proteins, the edges represented the protein-interactions, the larger and brighter the node was, the more count-numbers of proteins that interact with this node, and the thicker and brighter the edge was, the higher the value of Edgebetweenness in this edge.

The volcano-plot figures of the DEGs were listed on the left side of Fig 8A and 8B; after filtering, 536 of the major DEGs were detected in the comparison group of T_1/CT, and 818 were detected in the comparison group of T_48/T_1 (S7 Table).

Base on the STRING database, 175 protein pairs were obtained in the comparison group of T_1/CT (S8 Table), and 164 protein pairs were detected in the comparison group of T_48/T_1 (S9 Table). The main PPI network of T_1/CT was constructed; the “Heat shock 70kDa protein 8” (HSPA8), “Polymerase (RNA) II (DNA directed) polypeptide A” (POLR2A), “DEAH (Asp-Glu-Ala-His) box polypeptide 9” (DHX9), “Serine/arginine repetitive matrix 1” (SRRM1) and “Splicing factor 3a, subunit 2” (SF3A2) were detected as the top five proteins having the quantity of counted protein-interactions (Fig 8A, the right side). The main PPI network of T_48/T_1 was also formed; the HSPA8, SRRM1, “Serine/arginine-rich splicing factor 1” (SRSF1), “Serine/arginine repetitive matrix 2” SRRM2 and “Serine/arginine-rich splicing factor 4” (SRSF4) were discovered as the top five genes/proteins containing the number of counted protein-interaction (Fig 8B, the right side).

With the help of Cytoscape software, the top EdgeBetweenness values of the PPI networks were summarized and listed in Table 5, and the interaction of “EP300 (interacts with) HSPA8” contained the highest EdgeBetweenness value in the comparison group of T_1/CT and showed the hub protein-interaction in the network (Table 5). The interaction of “HSPA4 (interacts with) NUP54” contained the highest EdgeBetweenness value in the comparison group of T_48/T_1 and revealed the hub protein-interaction in the network (Table 5). The results indicated the important protein-interactions involved in the high-pH adaptation of *L*. *vannamei*.

**Table 5:** The important protein-interactions in the comparison groups of T_1/CT and T_48/T_1.

Comparison	Number	Protein-interaction	Experimental interaction ^a^	Combined score ^b^	EdgeBetweenness ^c^
T_1/CT	1	EP300 (interacts with) HSPA8	0.04	0.92	28.50
	2	DDX17 (interacts with) DHX9	0.65	0.83	27.50
	3	RTF1 (interacts with) POLR2A	0.61	0.98	26.00
	4	PWP1 (interacts with) DDX17	0.06	0.73	19.00
	5	POLR2A (interacts with) EP300	0.45	0.96	18.33
	6	HSPA8 (interacts with) HNRNPL	0.00	0.91	18.00
	7	HSPA8 (interacts with) SF3A2	0.77	0.98	18.00
	8	EP300 (interacts with) NFKB1	0.40	0.97	15.33
	9	SMARCA5 (interacts with) EP300	0.20	0.95	15.00
	10	DOT1L (interacts with) RTF1	0.00	0.63	14.00
	11	EGLN1 (interacts with) POLR2A	0.36	0.64	13.00
	12	MT-ATP6 (interacts with) CYCS	0.00	0.61	12.00
	13	WIPF2 (interacts with) SMARCA5	0.00	0.63	12.00
	14	POLR2A (interacts with) PPIG	0.39	0.65	12.00
	15	POLR2A (interacts with) SMARCA5	0.52	0.66	12.00
	16	DHX9 (interacts with) POLR2A	0.36	0.94	12.00
	17	SRRM2 (interacts with) RBM25	0.85	0.97	10.17
	18	MITF (interacts with) EP300	0.37	0.96	10.00
T_48/T_1	1	HSPA4 (interacts with) NUP54	0.054	0.908	21.00
	2	FOS (interacts with) HSPA4	0.054	0.75	19.50
	3	NUP54 (interacts with) SRSF1	0	0.908	18.00
	4	NPPA (interacts with) FOS	0	0.924	15.00
	5	EGR3 (interacts with) FOS	0.066	0.642	15.00
	6	CYCS (interacts with) HSPA4	0.043	0.711	13.00
	7	MT-ATP6 (interacts with) CYCS	0	0.608	12.00
	8	SRSF1 (interacts with) SUGP1	0	0.97	11.00
	9	NFKB1 (interacts with) HMOX1	0	0.64	11.00

^"a"^ indicated the experimentally determined interaction scores obtained from the STRING database;

^"b"^ represented the manageable scores for this pair of protein-interactions;

^"c"^ showed the EdgeBetweenness value obtained from the Cytoscape software.

The interactions were filtered by condition of EdgeBetweenness values ≥ 10.0 (Nearly top 10% of all EdgeBetweenness). CYCS: Cytochrome c, somatic; DDX17: DEAD (Asp-Glu-Ala-Asp) box helicase 17; DHX9: DEAH (Asp-Glu-Ala-His) box polypeptide 9; DOT1L: Disruptor of telomeric silencing 1-like; EGLN1: Egl nine homolog 1; EGR3: Early growth response 3; EP300: Histone acetyltransferase p300; FOS: FBJ murine osteosarcoma viral oncogene homolog; HMOX1: Heme oxygenase (decycling) 1; HNRNPL: Heterogeneous nuclear ribonucleoprotein L; HSPA4: Heat shock 70kDa protein 4; HSPA8: Heat shock 70kDa protein 8; MITF: Microphthalmia-associated transcription factor; MT-ATP6: Mitochondrially encoded ATP synthase 6; NFKB1: Nuclear factor of kappa light polypeptide gene enhancer in B-cells 1; NPPA: Natriuretic peptide A; NUP54: Nucleoporin 54kDa; POLR2A: Polymerase (RNA) II (DNA directed) polypeptide A; PPIG: Peptidylprolyl isomerase G (cyclophilin G); PWP1: Periodic tryptophan protein 1 homolog; RBM25: RNA binding motif protein 25; RTF1: Paf1/RNA polymerase II complex component, homolog; SF3A2: Splicing factor 3a, subunit 2; SMARCA5: SWI/SNF related, matrix associated, actin dependent regulator of chromatin, subfamily a, member 5; SRRM2: Serine/arginine repetitive matrix 2; SRSF1: Serine/arginine-rich splicing factor 1; SUGP1: SURP and G patch domain containing 1; WIPF2: WAS/WASL interacting protein family, member 2.

Based on Table 5 and on the PPI networks, the candidate hub proteins were summarized and displayed in Table 6. The POLR2A gene was discovered in six pairs of important hub protein-interactions (detailed information of the protein-interactions was listed in Table 5) in the T_1/CT comparison group, which indicated that this gene was one of the most important regulated genes in response to high-pH stress in *L*. *vannamei* (Tables 5 and 6; Fig 8A and 8B, the right side). The genes POLR2A, EP300, HSPA8, SMARCA5, DDX17, DHX9 and RTF1 were discovered as the hub regulation-genes in the comparison group of T_1/CT; the genes HSPA4, FOS, NUP54, SRSF1 and CYCS were detected as the hub regulation-genes in the comparison group of T_48/T_1 (Tables 5 and 6).

**Table 6:** Summary of the candidate hub-regulation genes.

Comparison	Name	Count number in protein-interaction	Edge number in PPI network
T_1/CT	POLR2A	6	18
	EP300	5	9
	HSPA8	3	19
	SMARCA5	3	3
	DDX17	2	8
	DHX9	2	14
	RTF1	2	2
T_48/T_1	HSPA4	3	9
	FOS	3	9
	NUP54	2	6
	SRSF1	2	12
	CYCS	2	5

CYCS: Cytochrome c, somatic; DDX17: DEAD (Asp-Glu-Ala-Asp) box helicase 17; DHX9: DEAH (Asp-Glu-Ala-His) box polypeptide 9; EP300: Histone acetyltransferase p300; FOS: FBJ murine osteosarcoma viral oncogene homolog; HSPA4: Heat shock 70kDa protein 4; HSPA8: Heat shock 70kDa protein 8; NUP54: Nucleoporin 54kDa; POLR2A: Polymerase (RNA) II (DNA directed) polypeptide A; RTF1: Paf1/RNA polymerase II complex component, homolog; SMARCA5: SWI/SNF related, matrix associated, actin dependent regulator of chromatin, subfamily a, member 5; SRSF1: Serine/arginine-rich splicing factor 1.

### Real-time PCR validation

**Fig 9:**
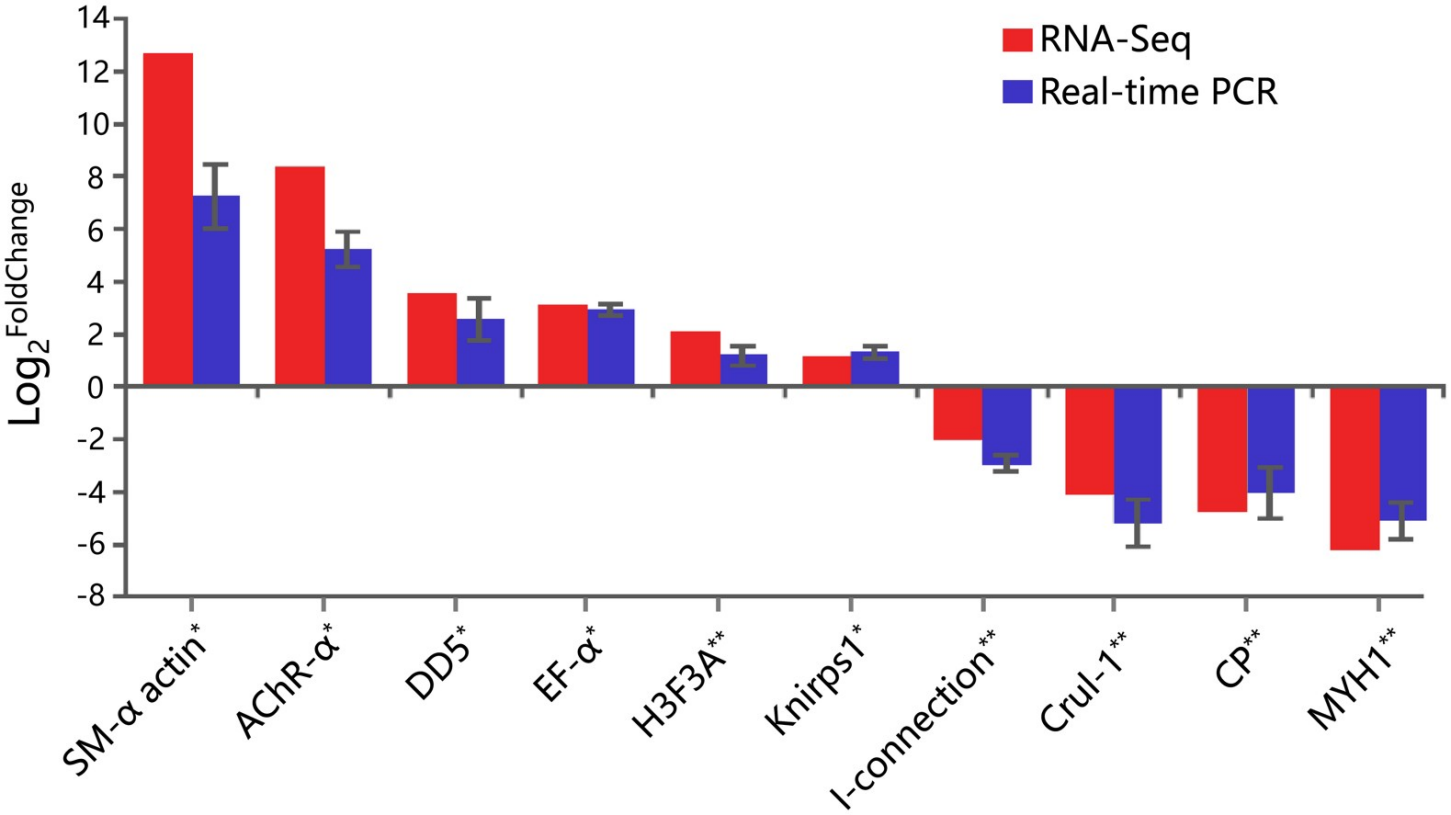
Validation for the 10 transcriptomic DEGs in the comparison groups of T_1/CT and T_48/T_1. Grouped results of the 10 genes; the blue columns indicated the real-time PCR results, and the red columns indicated the RNA-Seq results; the X axis indicated the gene names, and the Y axis represented the value of Log_2_^FoldChange^ value. The FoldChange values were obtained by the 2^–ΔΔCT^ method. Bars in this figure represented the standard deviation (SD). “*” represented the DEGs in the comparison group of T_1/CT, and “**” represented the DEGs in the comparison group of T_48/T_1. SM-α actin: Alpha smooth muscle actin; AChR-α: Acetylcholine receptor subunit alpha-like protein; DD5: Ubiquitin protein ligase E3 component n-recognin 5; EF-α: Elongation factor 1-alpha; H3F3A: H3 histone, family 3A; Knirps1: Transcriptional repressor 1; I-connectin: Invertebrate connectin, a giant spring-like protein; CruI-1: Magnesium-chelatase 30 kDa subunit (bchO); CP: Cuticular protein; MYH1: Myosin heavy chain type 1.

The treatment groups of CT, T_1 and T_48 were verified, and 10 genes were used to test the validations of the RNA-Seq results. The real-time PCR outcomes were summarized, and the grouped comparison results showed that all 10 candidate genes in the qPCR verification were consistent with the results of RNA-Seq technology (Fig 9).

## Discussion

The efficient of the transcriptome was verified in various studies [96]. According to the studies on *L*. *vannamei*, RNA-Seq had been applied to reveal several types of stress adaptation mechanisms [15, 17]. In the present study, the catalytic activity (GO:0003824) was the most annotated GO term and the signal transduction pathway was the most annotated Level-2 KEGG term in response to high-pH stress. cAMP is one of the most versatile second messengers, which mediates diverse cellular responses to extracellular signals [97]. Intracellular cAMP are induced by soluble adenylyl cyclases (sACs) and mainly effect the protein kinase A (PKA) for downstream regulation [97]. In the present study, cAMP signaling pathway was identified as the most annotated terms of signal transduction in KEGG pathway. The important role of cAMP in response to other types of stresses had been demonstrated in previous studies. The report of Zhao et al. (2016) proved that the cAMP concentrations of *L*. *vannamei* increased significantly and reached the maximum at 12 hours in low salinity (1.6%) environment [98]. Zhang et al. (2018) indicated that cAMP played a principal role in adapting to ammonia-N exposure in *L*. *vannamei* [99]. The cyclic nucleotide gated channel (CNGC), a type of component in the cAMP signaling pathway, was modulated by Ca^2+^-binding proteins and directly bound to cAMP to transduce the cellular signals for downstream regulation [100–101]. Hedgehog (Hh) signaling pathway played a central role in promoting the cellular processes and activities [102], and the aberrant activation of Hh signaling led to various types of cancer and defects in the organism cells [103]. The Ptc1 protein of cAMP signaling pathway could maintain the Hh pathway in an inactive state by inhibiting translocation of the signaling effector Smoothened (Smo) [104–105]. In this paper, the CNGCB1 (K04952) and Ptc1 (K06225) were detected as the major two parts KOs of the annotated components in cAMP signaling pathway. The results indicated that the components of CNGC might be involved in transducing the cellular signal of high-pH stress and Ptc1 might be participated to maintain the Hh pathway in a proper state for cell survival in high-pH environment of *L*. *vannamei*.

Transcription factors and kinases were two types of frequently traced categories involved in stress response studies [27, 106–107]. The kinase genes, mainly comprising MAPKs, Ser/Thr protein kinases and calcium-related signaling genes, were associated with the activity and coordination of the cell-expression network in response to extracellular stresses [17]. The signaling genes of transcription factors, Ser/Thr protein kinases, Ca^2+^ signaling pathway and MAPK cascades genes were summarized in the transcriptomic analysis of *Chrysanthemum nankingense* involved in low temperature tolerance [27]. In this study, the major signaling genes of transcription factors, Ser/Thr protein kinases and calcium-related signaling genes were detected; the MAPKs were not significantly transcribed in the adaptation of high-pH environment. Calcium impacted nearly every aspect of cellular life and fulfilled its key role in transferring extracellular stress signals into chromatin for regulation [17, 59, 108]. Ca^2+^ concentration was determined by feedback and feed-forward regulation of the involved transport proteins [109]. The CNGCs were nonselective cation channels that did not discriminate well between alkali ions and even pass divalent cations, in particular Ca^2+^ [100]. In the present study, the CNGC was suggested as the major annotated component in cAMP signaling pathway and the calcium-related signaling genes were detected as important type of signaling genes. Therefore, the results indicated that the molecular components of “CNGC-Ca^2+^” might play key role in transducing the high-pH stress signal in *L*. *vannamei*.

Crustaceans mainly depend on a non-specific immune system that includes circulating hemocytes and various active substances [98, 110]. Environmental stress might increase the vulnerability of shrimp to the pathogens normally present in the water by reducing the capacity of their immune responses [53, 80, 111]. In this paper, 17 types of major immune-related categories of DEGs were queried; after analyzing, the IgSF, HSP, GST, proPO/PO, SOD, ALF and lipoprotein were detected as the top 7 categories of expressing immune factors in response to high-pH stress in *L*. *vannamei*. The IgSFs were defined as molecules containing homologous domains and thought to play significant roles in innate defense in invertebrates [79, 112]. The IgG-like protein in *L*. *vannamei* was identified to contain the conserved domain with hemocyanin [112], and was involved in oxygen transporting and participating the antiviral and phenoloxidase-like activities [113], which indicated that the IgSFs might be one of the important types of immune factors for high-pH adaptation in *L*. *vannamei*. The GSTs were well known to alleviate the toxicity of diverse endogenous and exogenous compounds (xenobiotics) [114] through peroxidase and isomerase activities, biosynthesis of physiologically important biomolecules, as well as through the ligand binding (non-catalytic activity) [115–117], and possibly were involved in response to high-pH stress in *L*. *vannamei*. Our results indicated the major transcribed immune factors of *L*. *vannamei* in term of high-pH stress and the results would be benefit for future studies referring to the farming industry of this species in saline-alkali water areas.

The protein–protein interactions (PPIs) are the available bioinformatics analysis for computing the associations between genes and for predicting the key candidate genes and pathways [36, 118]. Yang et al. (2018) identified 3 candidate genes from 440 up- and 256 down-regulated DEGs as the potential hub regulation-genes involved in the acute phase of myocardial infarction [92]. In this paper, the PPI networks were constructed with 536 (T_1/CT comparison) and 818 (T_48/T_1 comparison) major DEGs, and the protein-interactions of “EP300-HSPA8” and “HSPA4-NUP54” were detected as the most important interactions in response to the high-pH stress. The important biological meanings of stress-induced protein-interactions raised by the present study had partly been interpreted in previous works. The acetyltransferase, EP300, was identified to control the cellular level of activatable heat shock transcription factor 1 (HSF1) in a manner linked with the clearance of misfolded proteins for adaptation to proteotoxic stress in HeLa cells [119]. Heat shock proteins could help to escape the mRNA export inhibition via recruiting nucleoporin-related proteins, and this biological interaction during stress conditions [120–121]. The results indicated that the PPIs analyzing might be available for computing and predicting the hub genes of *L*. *vannamei* in adapting to high-pH stress.

Heat shock proteins (HSPs) are defined as molecular chaperones, which play vital roles in maintaining protein homeostasis and in facilitating protein folding and degradation [122]. According to their monomeric molecular mass, HSPs are divided into HSP100, HSP90, HSP70, HSP60, HSP40 and small HSPs (sHSPs) [81, 122]. HSP70s are implicated in a wide variety of cellular processes via binding their substrate-binding domain to a short polypeptide with recognition motifs [123]. In the present paper, high-pH stress experiments were conducted on *L*. *vannamei*, the HSPs were detected as the main expressed genes among the major immune factors, and two HSP70 family genes (HSPA8 and HSPA4) were discovered as the hub regulation genes in the PPI networks. The key role of HSP70s had been proved involving in response to various stressful stimuli in previous studies [124–125]. The results of Duan et al. (2018) showed that the expression of HSP70 increased to the highest level by exposing the *L*. *vannamei* to nitrite stress for 6 hours [126]. Western blot analysis in the gill tissue of *L*. *vannamei* indicated that the expression of HSP70 was up-regulated by infecting the pathogens of white spot syndrome (WSSV) and hypodermal-hematopoietic necrosis (IHHNV) [127]. The mRNA and protein expression levels of HSP70 were observed highest in the heat-stress group of goats (*Capra hircus*) [128]. These results indicated that HSP70s might play essential important roles in the adaptation of high-pH stress in *L*. *vannamei*.

In conclusion, the first transcriptome analysis involved in high-pH stress was conducted on gill tissues of *L*. *vannamei*, the major high-pH associated immune factors and pathways were identified, PPI networks of major DEGs were constructed, and the hub regulation-genes were predicted. Our results provided the expression profiles and potential candidate hub genes for revealing the regulation mechanism in *L*. *vannamei* in response to high-pH stress. The construction of a PPI network was a useful method for further understanding and for revealing the hub regulation-genes in response to abiotic stress in the shrimp species.

## Supporting information

S1 File The complete nucleotide sequences of each Unigene. (TXT)

S1 Table The primers used for real-time PCR. (XLSX)

S2 Table The DEG information for the cluster of orthologous groups. (XLSX)

S3 Table Annotated pathway categories in Signal transduction of Level 2 KEGG. (XLSX)

S4 Table Annotated categories and DEGs in cAMP signaling pathway. ^a^ The numbers in the parentheses indicated the Log_2_^FoldChange^ value.
(XLSX)

S5 Table The major differentially-expressed immune factors. FPKM: metric fragments per kilobase of transcript per million mapped reads.
(XLSX)

S6 Table The major differentially-expressed signaling genes. FPKM: metric fragments per kilobase of transcript per million mapped reads.
(XLSX)

S7 Table The major differentially-expressed genes among the comparisons of T_1/CT and T_48/T_1. "-" represented absence of this unigene in the related group of transcript. FPKM: metric fragments per kilobase of transcript per million mapped reads.
(XLSX)

S8 Table Protein interactions obtained from the STRING database of the comparisons of T_1/CT. (XLSX)

S9 Table Protein interactions obtained from the STRING database of the comparisons of T_48/T_1. (XLSX)
